# SG-APSIC1162: Challenges in building and running a 4,000-bed COVID-19 intensive care unit in an exhibition center

**DOI:** 10.1017/ash.2023.19

**Published:** 2023-03-16

**Authors:** Martin Kiernan

**Affiliations:** Richard Wells Research Centre, University of West London, London, United Kingdom

## Abstract

**Objectives:** To describe the design process for a hospital in an exhibition center. We discuss challenges during the building process and areas in which risk assessments had to be made and practices modified to mitigate suboptimal conditions. **Methods:** UK National Health Service designers and military planners worked in conjunction with the infection prevention and control team (IPCT) to work with the existing infrastructure. The clinical area was deemed to be an aerosol-generating procedure (AGP) zone because it was entirely an intensive care unit. The challenges included no oxygen line, a lack of hot water, minimal access to cold water, almost no drainage, and a lack of physical space in which to carry out many necessary procedures. These challenges were overcome either by design or by changes to usual practices through mitigation measures. The IPCT had key roles in ensuring staff and patient safety and personal protective equipment (PPE) inventory management as well as donning and doffing procedures. **Results:** The Nightingale Hospital became a fully functioning ICU within 10 days of the build commencing, and the first patients were admitted within a few days. The hospital was used only sparingly because the national pandemic lockdown was in effect. In total, 72 patients were admitted, with a survival rate of 63%, comparable to established ICUs. Transmission rates of COVID-19 in staff were very low among those working clinically. The unit closed in June 2020 but reopened in January 2021 for rehabilitation with a smaller number of beds but better facilities as a result of our experience in the first iteration. **Conclusions:** A temporary hospital was built in an exhibition center to successfully manage a number of patients. Even in a temporary hospital facility that was limited in services, successful outcomes were achieved.

